# PB.12. Audit and root-cause analysis of classification 2 and 3 interval cancers

**DOI:** 10.1186/bcr3703

**Published:** 2014-11-03

**Authors:** D Cox, A Powell

**Affiliations:** 1 Sandwell & West Birmingham Hospitals NHS Trust, Birmingham, UK

## Introduction

The definition of an interval cancer is a cancer diagnosed between scheduled screening rounds. Interval cancer rates are an indication of the effectiveness of a Breast Screening Service in achieving early cancer detection, thus reducing overall mortality secondary to breast cancer, and represent a National Health Service Breast Screening Programme (NHSBSP) standard. The British Society of Breast Radiology (BSBR) Interval Classifications are as follows: 0 = unclassifiable, 1 = normal/benign, 2 = uncertain and 3 = suspicious. An audit was undertaken of interval cancers assigned a classification of 2 or 3 within our Breast Service over a 3-year period with the aim of determining and implementing improved clinical practice in a learning environment.

## Methods

The screening and symptomatic records were collected for all NHSBSP clients over a 3-year period subsequently diagnosed with a classification 2 or 3 interval cancer to establish the interval cancer rate benchmarked against NHSBSP standards and to determine the causal factors within the screening pathway.

## Results

During the audit period 93,296 women underwent breast screening. The total number of interval cancers was 220. Of these 52 were assigned a 2 or 3 interval cancer classification. The NHSBSP standard of 1.2 interval cancers per 1000 women screened for the first 2 years following screening was not achieved for women screened within the initial 2 years of the audit period.

## Conclusion

Causal factors were most commonly associated with double mammographic screen reading. Subsequent interval cancers occurring most commonly during an incident screen in women aged 50 to 59 years, 12 to 24 months following NHSBSP screening.

